# Cerebrovascular carbon dioxide reactivity is intact in chronic kidney disease

**DOI:** 10.14814/phy2.15998

**Published:** 2024-04-03

**Authors:** Justin D. Sprick, Jeann Sabino‐Carvalho, Elsa Mekonnen, Melissa McGranahan, Matias Zanuzzi, Dana DaCosta, Jeanie Park

**Affiliations:** ^1^ Department of Kinesiology, Health Promotion and Recreation University of North Texas Denton Texas USA; ^2^ Division of Renal Medicine, Department of Medicine Emory University School of Medicine Atlanta Georgia USA; ^3^ Research Service Line, Atlanta VA Health Care System Decatur Georgia USA

**Keywords:** cerebral blood flow, cerebrovascular disease, renal disease

## Abstract

Chronic kidney disease (CKD) is characterized by an elevated risk for cerebrovascular disease including stroke. One mechanism that may contribute to this heightened risk is an impairment in cerebrovascular carbon dioxide reactivity (CVR). We compared CVR between CKD patients stages III–IV and controls (CON) without CKD but matched for hypertension and diabetes status. CVR was measured via 5% CO_2_ inhalation followed by voluntary hyperventilation in 14 CKD and 11 CON participants while mean arterial pressure, end‐tidal carbon dioxide, and middle cerebral artery blood velocity (MCAv) were measured continuously. CVR was quantified as the linear relationship between etCO_2_ and MCAv. We observed no difference in CVR between groups. Hypercapnic CVR: CKD = 1.2 ± 0.9 cm/s/mm Hg, CON = 1.3 ± 0.8 cm/s/mm Hg, hypocapnic CVR: CKD = 1.3 ± 0.9 cm/s/mm Hg, CON = 1.5 ± 0.7 cm/s/mm Hg, integrated CVR: CKD = 1.5 ± 1.1 cm/s/mm Hg, CON = 1.7 ± 0.8 cm/s/mm Hg, *p* ≥ 0.48. Unexpectedly, CVR was inversely related to estimated glomerular filtration rate in CKD (*R* ^2^ = 0.37, *p* = 0.02). We report that CVR remains intact in CKD and is inversely related to eGFR. These findings suggest that other mechanisms beyond CVR contribute to the elevated stroke risk observed in CKD.

## 1 INTRODUCTION

Patients with chronic kidney disease (CKD) have a substantially elevated risk for cerebrovascular disease including stroke (Lee et al., 2010). Additionally, when stroke does occur, CKD patients suffer from poorer recovery (Kumai et al., 2012) and higher mortality rates (Tsagalis et al., 2009). One mechanism that may contribute to this heightened risk and poor prognosis is an impairment in cerebrovascular carbon dioxide reactivity (CVR), the mechanism through which cerebral vessels dilate and constrict in response to fluctuations in arterial carbon dioxide tension (PaCO_2_). CVR is critically important for the maintenance of cerebral acid–base balance and is widely recognized as a key mechanism governing cerebral blood flow regulation (Hoiland et al., 2019).

CVR is impaired in other chronic disease states such as heart failure, diabetes, and atrial fibrillation (Georgiadis et al., 2000; Junejo et al., 2019; Kadoi et al., 2003) and is predictive of future stroke in high‐risk groups such as patients with carotid artery stenosis (Markus & Cullinane, 2001; Reinhard et al., 2014; Silvestrini et al., 2000). Despite the known link between CVR and stroke, very few investigations have examined CVR in the setting of kidney disease. With the exception of one recent report that CVR remains intact in CKD compared to a completely healthy control group (Oh et al., 2023), most prior studies have focused almost exclusively on patients with end‐stage kidney disease (ESKD) receiving renal replacement therapy (Ishida et al., 2018; Kuwabara et al., 2002; Szprynger et al., 2000). It is unclear if these small studies in ESKD can be extrapolated to CKD patients not on renal replacement therapy, and whether CVR is altered in early stages of kidney disease. We aimed to determine if CVR is impaired in patients with CKD stages III–IV (moderate to severe CKD) compared to an age and comorbidity‐matched control (CON) group free from renal disease but matched for diabetes and hypertension status, the two leading causes of CKD.

## 2 METHODS

### 2.1 Participants

Sedentary individuals with CKD stages III–IV (eGFR between 15 and 59 mL/min/1.73 m^2^), as defined by the race‐free CKD‐EPI equation (Inker et al., 2021) and CON participants without CKD were recruited from outpatient nephrology clinics and family medicine clinics in the Atlanta or Dallas Fort Worth Metropolitan areas for participation in this study. All clinical diagnoses (i.e., CKD, hypertension, and diabetes) were made by a physician. All CKD participants had stable kidney function with no greater than 20% fluctuation in eGFR over the prior 3 months. Exclusion criteria for both groups included uncontrolled hypertension (blood pressure > 160/90 mm Hg), vascular disease (including prior stroke), clinical evidence of heart failure or heart disease determined by electrocardiogram (ECG) or echocardiogram, engagement in regular exercise (defined as greater than 20 min of physical activity at least twice per week over the last 6 months), drug or alcohol use disorder within the past 12 months, and pregnancy.

### 2.2 Instrumentation

Participants reported to the Human Physiology Laboratory at Emory University School of Medicine or the Applied Physiology Laboratory at the University of North Texas after abstaining from food, alcohol, and caffeine for at least 12 h, and exercise for a minimum of 24 h. Participants taking medications took all medications as prescribed. Resting blood pressure and heart rate (Omron, Hem907XL, Hoffman Estates, IL) were measured in triplicate in the seated position after 5 min of quiet rest with the arm supported at the heart level in accordance with the American College of Cardiology/American Heart Association Guidelines (Whelton et al., 2018). A 10 mL blood sample was collected via venipuncture for measurement of serum creatinine.

Following the resting measurement, participants were positioned supine and instrumented for continuous measurement of heart rate (HR) via a standard three lead ECG (AD Instruments, Bella Vista, NSW, Australia), and beat‐to‐beat arterial pressure via finger photoplethysmography (Finometer, Finapress Medical Systems, Amsterdam, The Netherlands) applied to the dominant hand. Respiration rate and end‐tidal CO_2_ (etCO_2_) were measured on a breath‐by‐breath basis through an oral‐nasal cannula via capnography (ML206, AD Instruments, Bella Vista, NSW, Australia). Middle cerebral artery blood velocity (MCAv) was measured via transcranial Doppler (TCD) ultrasound (Multi‐Dop, DWL, Cameron Park, CA). Specifically, 2 MHz probes were secured over the temporal window of the participants head via adjustable headgear (Diamon, DWL, Cameron Park, CA) while MCAv was acquired using a standardized approach as outlined in the literature (Willie et al., 2011). Efforts were made to ensure that MCAv recordings were obtained from the left side of the head in all participants; however, the side with the best signal quality was used for analysis. Participants were equipped with a facemask attached to a gas cylinder containing 5% CO_2_, 21% O_2_ and balanced with nitrogen for the subsequent CVR assessment outlined in the experimental protocol.

### 2.3 Experimental protocol

The following experimental protocol was used to measure CVR at both data collection sites. A 10‐min resting baseline was observed to allow all hemodynamic and cerebrovascular parameters to stabilize. Participants were paced to breathe at a frequency of 12 breaths/min throughout baseline and the subsequent assessment of CVR. Hypercapnia was then achieved by increasing the flow of the gas tank until etCO_2_ increased by 5 mm Hg from resting values over a period of 2 min. A 30‐s washout was then observed. Next, participants were instructed to gradually increase their tidal volume while maintaining their paced respiration rate of 12 until etCO_2_ was reduced by ‐5 mm Hg from resting values over a period of 2 min. The entire CVR assessment was completed in 270 s. This protocol has previously been used to assess CVR in both healthy (Fan et al., 2019; Peebles et al., 2012) and clinical (Fan et al., 2020) populations.

### 2.4 Data analysis

All continuous waveform data (e.g., ECG, arterial pressure, MCAv, respiratory rate, etCO_2_) were collected at 1000 Hz (Labchart, AD Instruments, Bella Vista, NSW, Australia) and analyzed offline via specialized software (Ensemble, Elucimed, Wellington, New Zealand). The timing of each cardiac cycle was determined from the R‐wave of the ECG signal, and beat‐to‐beat arterial pressures and MCAv velocities were then detected. Mean arterial pressure (MAP) and mean MCAv were automatically calculated as the area under the arterial pressure and cerebral blood velocity waveform. The etCO_2_ tracing was left shifted by ‐3 s to account for inherent delays in the sampling line. Baseline values for all measured variables were averaged over the 5‐min period prior to assessment of CVR.

CVR (cm/s/mm Hg) was quantified as the linear relationship between MCAv and etCO_2_ on a breath‐by‐breath basis while accounting for MAP as a covariate. The etCO_2_ trace was shifted relative to both arterial pressure and MCAv signals. The time interval corresponding to the maximum positive cross correlation between the MCAv and etCO_2_ signals was identified, and time shifted to account for the physiologic latency associated with the arterial transit time of CO_2_ (Fan et al., 2019, 2020; Peebles et al., 2012). This analysis was automatically performed using specialized software (Ensemble, Elucimed, Wellington, New Zealand). To confirm the presence of kidney disease in the CKD group and ensure that the CON group was free from renal disease, eGFR was calculated based on the serum creatinine measurement using the CKD‐EPI equation (Inker et al., 2021).

### 2.5 Statistical analysis

A power analysis was performed based on cross‐sectional studies comparing CVR between healthy adults and related clinical populations (Georgiadis et al., 2000; Slessarev et al., 2021) as well as our own preliminary data in CKD. This analysis indicated that with *α* = 0.05 and *β* = 0.8, a sample size of *N* = 11/group would provide power to detect a 1 cm/s/mm Hg difference (SD = 0.8) in integrated CVR between groups. All data are presented as mean ± SD. Normality was assessed using the Shapiro–Wilk test. Participant demographic data, medication use, resting hemodynamics, and cerebrovascular parameters were compared between groups via unpaired, two‐tailed, *t*‐tests for continuous variables, or chi‐square analysis for categorical variables. Hypercapnic, hypocapnic, and integrated (hyper‐ + hypocapnic) CVR was compared between groups via unpaired, two‐tailed, *t*‐tests. Linear regression was used to examine the relationship between CVR and eGFR.

## 3 RESULTS

### 3.1 Participants

Fourteen patients with CKD stages III‐IV and 11 controls without kidney disease were recruited to participate in this study. Demographic data for both groups are reported in Table 1. There were no differences in age, sex, weight, blood pressure, or the presence of diabetes or hypertension between groups. There were more Black participants in the CKD group compared to the CON group (*p* = 0.01). Use of anti‐hypertensive medication was common in both groups, with more CON participants taking angiotensin converting enzyme inhibitors/angiotensin receptor blockers (ACEi/ARB) versus the CKD group (*p* = 0.01).

**TABLE 1:** Participant demographic data and medication use.

Characteristic	CKD	CON	*p*
*n*	14	11	
Age (year)	59 ± 15	61 ± 6	0.62
eGFR (mL/min/1.73 m^2^)	40 ± 11	79 ± 16	0.003
Systolic arterial pressure	124 ± 17	136 ± 14	0.09
Diastolic arterial pressure	78 ± 12	85 ± 12	0.15
Sex (M/F)	7/7	6/5	0.82
Race, *n* (%)			0.01
Black	11 (79%)	3 (27%)	
White	3 (21%)	8 (73%)	
Body weight (kg)	82 ± 18	89 ± 21	0.39
Body mass index (kg m^−2^)	29 ± 7	32 ± 6	0.35
Diabetes, *n* (%)	5 (36%)	3 (27%)	0.65
Hypertension, n (%)	11 (79%)	10 (91%)	0.40
Anti‐hypertensive medications, *n* (%)			
Calcium channel blockers, *n* (%)	8 (57%)	4 (36%)	0.30
ACE inhibitors/ARBs, *n* (%)	6 (43%)	10 (91%)	0.01
Diuretics, *n* (%)	5 (36%)	4 (36%)	0.97
β‐blockers, *n* (%)	5 (36%)	3 (27%)	0.65
Statins, *n* (%)	7 (50%)	4 (36%)	0.50

Abbreviations: ACE, angiotensin converting enzyme; ARB, angiotensin receptor blocker; CKD, chronic kidney disease; CON, control; eGFR, estimated glomerular filtration rate.

### 3.2 Baseline hemodynamics and cerebrovascular parameters

Resting hemodynamic and cerebrovascular parameters are provided in Table 2. There were no differences in resting heart rate, arterial pressure, MCAv, or etCO_2_ between groups (*p* ≥ 0.07). Blood pressure measurements are elevated in both groups compared to resting values likely due to methodological differences in how the measurement was performed (Oscillometric cuff vs finger photoplethysmography).

**TABLE 2:** Resting hemodynamic and cerebrovascular parameters.

Characteristic	CKD	CON	*p*
*n*	14	11	
Heart rate (bpm)	68 ± 14	66 ± 9	0.71
Systolic blood pressure (mm Hg)	140 ± 19	141 ± 20	0.90
Diastolic blood pressure (mm Hg)	72 ± 11	81 ± 12	0.07
Mean arterial pressure (mm Hg)	99 ± 12	106 ± 17	0.20
Mean middle cerebral artery blood velocity (cm/s)	53 ± 18	46 ± 13	0.33
Cerebrovascular resistance index (mm Hg/cm/s)	2.0 ± 0.6	2.5 ± 0.7	0.13
End‐tidal partial pressure of carbon dioxide (mm Hg)	36 ± 4	36 ± 4	0.85

Abbreviations: CKD, chronic kidney disease; CON, Control.

### 3.3 Cerebrovascular reactivity

**FIGURE 1:**
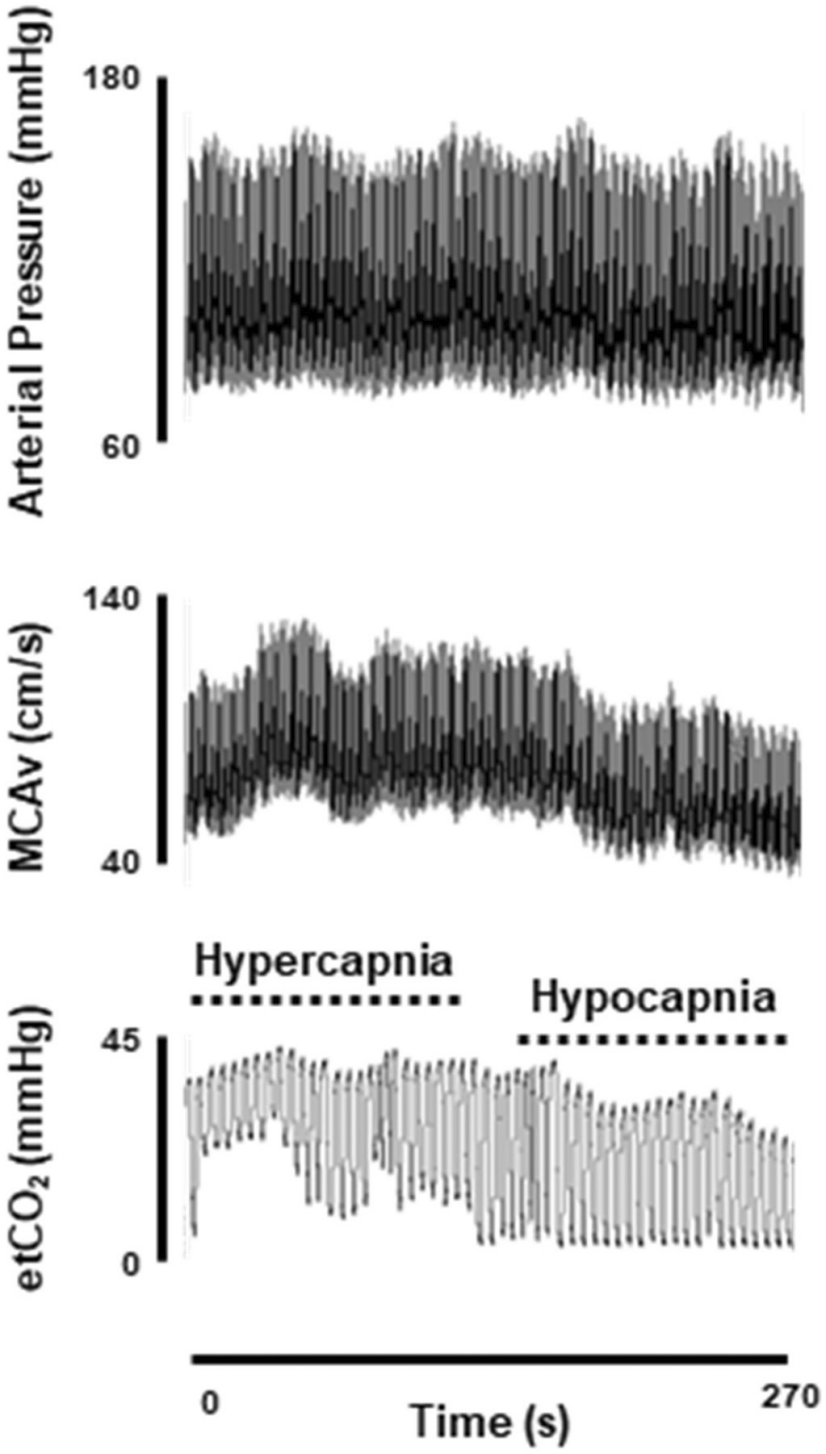
Representative arterial pressure (top), middle cerebral artery blood velocity (middle), and end‐tidal carbon dioxide (bottom) recordings during assessment of cerebrovascular carbon dioxide reactivity in a chronic kidney disease participant.

**FIGURE 2:**
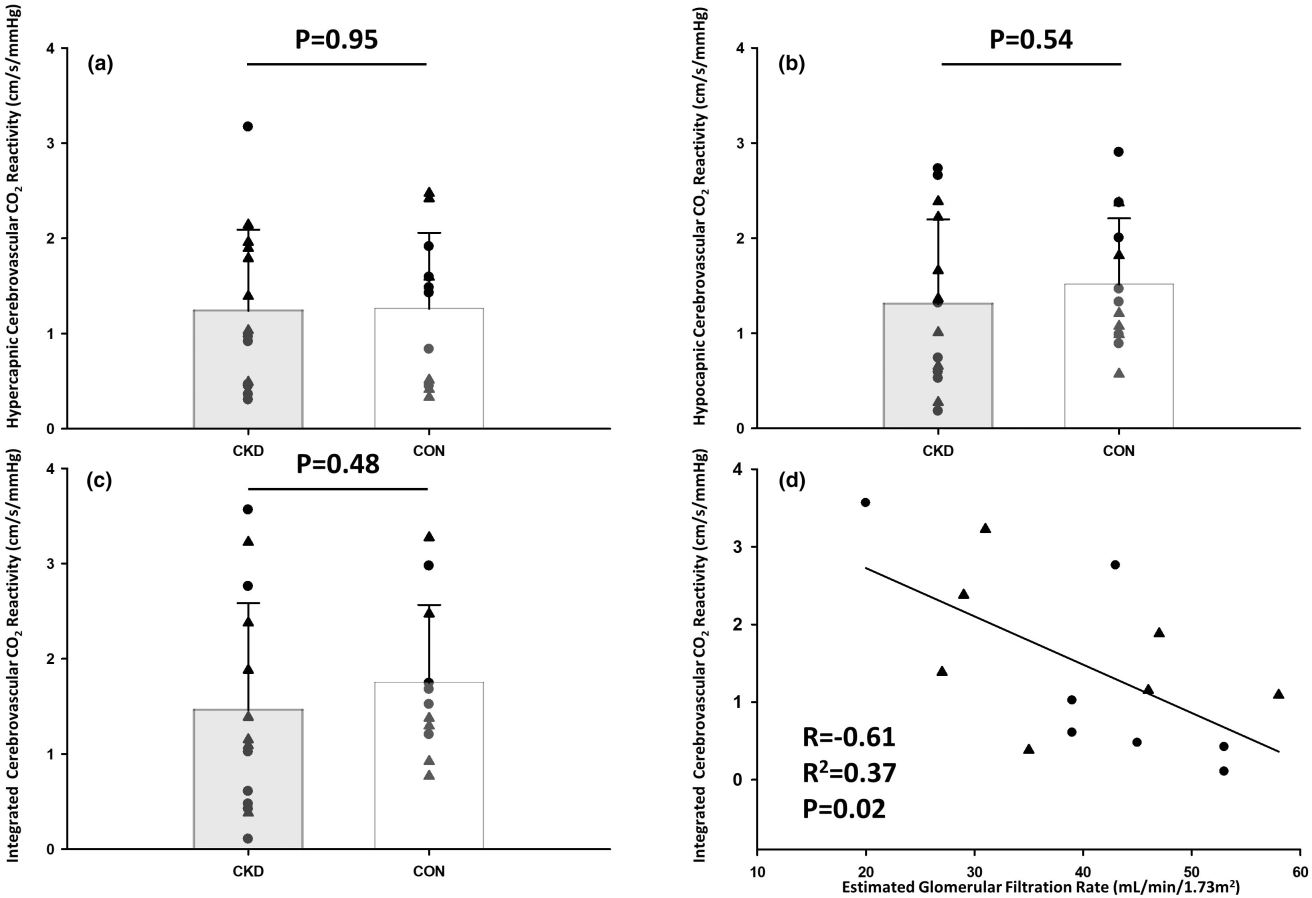
Hypercapnic (a), hypocapnic (b), and integrated (c) cerebrovascular carbon dioxide reactivity (CVR) in N = 14 CKD (7 M/7F) and N = 11 CON (6 M/5F). Hyper‐, hypo‐, and integrated CVR were compared between groups via two‐tailed, unpaired t‐tests. *p* ≥ 0.48 for all group comparisons. Linear relationship between cerebrovascular carbon dioxide reactivity and estimated glomerular filtration rate in chronic kidney disease participants (d). The relationship between integrated cerebrovascular carbon dioxide reactivity and estimated glomerular filtration rate was assessed via linear regression (*p* = 0.02). Triangles represent male participants. Circles represent female participants.

MCAv and etCO_2_ increased during 5% CO_2_ inhalation and decreased during voluntary hyperventilation for all participants. Figure 1 shows a representative recording for arterial pressure, MCAv, and etCO_2_ during the CVR protocol in a CKD participant. Hypercapnic, hypocapnic, and integrated CVR are shown in Figure 2. There were no differences in CVR between groups. Hypercapnic CVR: CKD = 1.2 ± 0.9 cm/s/mm Hg, CON = 1.3 ± 0.8 cm/s/mm Hg (Panel A), hypocapnic CVR: CKD = 1.3 ± 0.9 cm/s/mm Hg, CON = 1.5 ± 0.7 cm/s/mm Hg (Panel B), and integrated CVR: CKD = 1.5 ± 1.1 cm/s/mm Hg, CON = 1.7 ± 0.8 cm/s/mm Hg (Panel C), *p* ≥ 0.48. Within the CKD group, CVR was inversely related to eGFR (*R* ^2^ = 0.37, *p* = 0.02, Panel D). However, there was no relationship between CVR and eGFR for the CON group (*p* = 0.70).

## 4 DISCUSSION

In this investigation, we demonstrate that CVR remains intact in patients with CKD stages III‐IV in comparison with CON group matched for hypertension and diabetes status but without kidney disease. In addition, we unexpectedly observed an inverse relationship between CVR and eGFR in CKD. These findings are clinically relevant for stroke prevention in CKD and suggest that other mechanisms beyond CVR contribute to the heightened cerebrovascular disease burden experienced in CKD.

To the best of our knowledge, this is the first investigation to compare CVR between CKD patients stages III‐IV and controls matched for diabetes and hypertension status. Most prior studies examining CVR in renal disease have focused on ESKD (Ishida et al., 2018; Kuwabara et al., 2002; Szprynger et al., 2000). One recent investigation compared hypercapnic CVR between CKD patients stages III‐IV and a completely healthy control group, and similarly observed no differences between groups (Oh et al., 2023). Our findings agree with this report and further demonstrate that CVR is maintained in CKD in comparison with a control group matched for diabetes and hypertension, and that in addition to hypercapnic CVR, hypocapnic CVR also remains unaltered in CKD.

We report the novel finding that CVR is inversely related to eGFR within CKD. Although we observed no differences between groups, these findings suggest that reductions in kidney function are associated with enhanced CVR within CKD. One prior study similarly reported enhanced CVR in pediatric patients with chronic renal failure in comparison with healthy children with normal renal function. It was speculated that this cerebral hyperreactivity could be the result of impaired cerebral autoregulation (Szprynger et al., 2000); however, we have recently demonstrated that cerebral autoregulation remains intact in adults with CKD (Sprick et al., 2022) so this is an unlikely explanation for our findings. Other factors within CKD that could mediate the association between renal insufficiency and enhanced CVR include metabolic acidosis and anemia. Healthy adults exhibit enhanced CVR during ascent to high altitude due to respiratory alkalosis with subsequent renal compensation (Ainslie et al., 2012). Reductions in serum bicarbonate concentrations due to metabolic acidosis may thus artificially enhance CVR in CKD. Hemoglobin is inversely related to peripheral vascular function in CKD (Yilmaz et al., 2009) so it is possible that anemia may also enhance CVR in CKD. These possibilities should be explored in future studies.

There are several methodological considerations that should be considered as they relate to the interpretation of these findings. First, we measured MCAv velocity via TCD which relies on the assumption that MCA diameter remains constant. However, MCA diameter may change during pronounced hyper‐ or hypocapnia (Coverdale et al., 2014; Verbree et al., 2014) in which case TCD‐derived measures of MCAv would underestimate true changes in cerebral blood flow. However, recent work has shown that changes in MCA diameter during fluctuations in PaCO_2_ are largely protocol‐dependent (Al‐Khazraji et al., 2021), and are likely minimized by the use of a ramp, breath‐by‐breath, CVR protocol such as the one used in the current investigation. Second, a greater proportion of the CON group was taking ACEi/ARB vs the CKD group. Prior work has shown that ARB usage preserves CVR over a 12‐month period in hypertensive adults, so it is possible that ACEi/ARB use in the CON group may have enhanced CVR (Hajjar et al., 2013). Given that CVR was inversely related to eGFR in CKD, this enhancement may have potentially masked group differences. Additionally, given the relatively small sample size, we were underpowered to examine sex differences in CVR in CKD. Sex differences in CVR are present in healthy adults (Kastrup et al., 1997), and sex differences in peripheral microvascular function are present in CKD (Kirkman et al., 2022). Future work should examine whether sex differences in CVR are also present in CKD. Lastly, there was a greater proportion of black participants in the CKD group vs the CON group. Although racial differences in CVR have been relatively understudied, one recent investigation demonstrated no differences in CVR between young black and white women, despite differences in peripheral vascular function (Martin et al., 2023), thus while it is unlikely that racial differences contributed to our findings, future work should examine whether racial differences in CVR are present in patients with CKD.

## 5 CONCLUSION

In conclusion, we observed no impairment in CVR during 5% CO_2_ inhalation and voluntary hyperventilation in patients with CKD stages III‐IV compared to age‐ and comorbidity‐matched controls. However, within patients with CKD, CVR is inversely related to eGFR. These findings suggest that other mechanisms likely contribute to the increased cerebrovascular disease risk exhibited in CKD.

## 6 PERSPECTIVES AND SIGNIFICANCE

Patients with CKD exhibit an elevated risk for cerebrovascular disease which may be mediated, in part, by impairments in cerebral blood flow regulation. We demonstrate that CVR is intact in CKD stages III‐IV suggesting that other factors contribute to the heightened stroke risk observed in CKD.

## FUNDING INFORMATION

This work was supported by the American Heart Association (Career Development Award #929040, PI: Sprick), and NIH grants R01HL135183 and R33AT010457.

## ETHICS STATEMENT

This study was approved by the University of North Texas Institutional Review Board, the Atlanta Veteran's Affairs (VA) Health Care System, and Emory University Institutional Review Board. Written informed consent was obtained for all study participants, and all study procedures conformed to the standards set forth by the Declaration of Helsinki.

## INSTITUTION WHERE WORK WAS PERFORMED

The Applied Physiology Laboratory, Department of Kinesiology, Health Promotion and Recreation, University of North Texas, 1921 Chestnut St, Denton, Texas, 76,201 & The Human Physiology Laboratory, Division of Renal Medicine, Emory University, 1639 Pierce Drive, Woodruff Memorial Research Building, 3300, Atlanta, Georgia, 30,322.

## Data Availability

The data sets generated during this study are available from the corresponding author upon reasonable request.
